# Protocol for a randomized controlled study of Iyengar yoga for youth with irritable bowel syndrome

**DOI:** 10.1186/1745-6215-12-15

**Published:** 2011-01-18

**Authors:** Subhadra Evans, Laura Cousins, Jennie CI Tsao, Beth Sternlieb, Lonnie K Zeltzer

**Affiliations:** 1 Pediatric Pain Program, Department of Pediatrics, David Geffen School of Medicine, University of California, Los Angeles, USA

## Abstract

**Introduction:**

Irritable bowel syndrome affects as many as 14% of high school-aged students. Symptoms include discomfort in the abdomen, along with diarrhea and/or constipation and other gastroenterological symptoms that can significantly impact quality of life and daily functioning. Emotional stress appears to exacerbate irritable bowel syndrome symptoms suggesting that mind-body interventions reducing arousal may prove beneficial. For many sufferers, symptoms can be traced to childhood and adolescence, making the early manifestation of irritable bowel syndrome important to understand. The current study will focus on young people aged 14-26 years with irritable bowel syndrome. The study will test the potential benefits of Iyengar yoga on clinical symptoms, psychospiritual functioning and visceral sensitivity. Yoga is thought to bring physical, psychological and spiritual benefits to practitioners and has been associated with reduced stress and pain. Through its focus on restoration and use of props, Iyengar yoga is especially designed to decrease arousal and promote psychospiritual resources in physically compromised individuals. An extensive and standardized teacher-training program support Iyengar yoga's reliability and safety. It is hypothesized that yoga will be feasible with less than 20% attrition; and the yoga group will demonstrate significantly improved outcomes compared to controls, with physiological and psychospiritual mechanisms contributing to improvements.

**Methods/Design:**

Sixty irritable bowel syndrome patients aged 14-26 will be randomly assigned to a standardized 6-week twice weekly Iyengar yoga group-based program or a wait-list usual care control group. The groups will be compared on the primary clinical outcomes of irritable bowel syndrome symptoms, quality of life and global improvement at post-treatment and 2-month follow-up. Secondary outcomes will include visceral pain sensitivity assessed with a standardized laboratory task (water load task), functional disability and psychospiritual variables including catastrophizing, self-efficacy, mood, acceptance and mindfulness. Mechanisms of action involved in the proposed beneficial effects of yoga upon clinical outcomes will be explored, and include the mediating effects of visceral sensitivity, increased psychospiritual resources, regulated autonomic nervous system responses and regulated hormonal stress response assessed via salivary cortisol.

**Trial registration:**

ClinicalTrials.gov NCT01107977.

## Introduction

### The impact of IBS

IBS is a complex, chronic, functional disorder for which there is no cure. The condition is characterized by abdominal pain/discomfort and altered bowel habits; other symptoms may include nausea, vomiting, and bloating. IBS prevalence estimates in North America for adults and adolescents are between 10 -15% [1,2]. Boys and girls appear to be equally affected until late adolescence [3], when sex differences emerge and women are twice as likely to be affected as men [4]. Direct costs of IBS to the U.S. health system are in the region of $10 billion, with indirect costs contributing a further $20 billion [5]. IBS and related functional bowel disorders have been ranked as a common cause of illness-related absenteeism, second only to the common cold [6]. In addition to increased healthcare use and absenteeism, IBS has been related to an elevated risk of other somatic intestinal and extraintestinal comorbidities, psychological comorbidities such as anxiety and depression [7], and to reduced quality of life [8].

There are no definitive biochemical, structural or serologic markers to assist diagnosis or assessment of treatment efficacy [9]. Currently, symptom-based approaches are used in diagnosis and the Rome criteria, now Rome III [10], is the current gold standard of diagnostic tools. The Rome diagnostic criteria presume the absence of a structural or biochemical explanation for the symptoms, and diagnosis is made by an experienced clinician. ROME III criteria consist of recurrent abdominal pain or discomfort for at least 3 months, onset at least 6 months previously and with at least two of the following: improvement with defecation, onset associated with a change in frequency of stool, and/or onset associated with a change in appearance of stool. Sub-categories are based on stool consistency as IBS with constipation, IBS with diarrhea, or IBS with mixed or alternating constipation and diarrhea [11]. Criteria exist for both children (4-18 years of age) and adults (over 18 years of age).

Although IBS is typically diagnosed in adults between 30 and 50 years of age [12], symptoms generally present earlier, beginning in childhood and adolescence and continuing through to adulthood [13]. Adolescence represents a particularly important time in the development of IBS symptoms. Prevalence estimates suggest an increase from around 8% in middle school to 14% in high school-aged students- a prevalence rate similar to that seen in adult populations [3]. Given the often protracted course of IBS, it is important to study the emergence of IBS and treatment options in young populations. Quality of life is also greatly compromised in young IBS sufferers. Varni and colleagues [14] found that IBS significantly affected physical, emotional, social, and school functioning in children and adolescents aged 5-18 years. In this study, children and adolescents were more likely to miss school, spend days sick in bed, be too ill to play, and need someone to care for them than were healthy children.

It is likely that IBS takes a particular toll on the school and social functioning of adolescents and early adults, when dating, social acceptance and academic performance become paramount concerns. Fear of pain, bloating, and/or diarrhea may severely limit the adolescent patient's willingness to attend school and social outings. Yet engagement in such activities may be vital for future functioning. Perceived academic competence has been found to moderate the relationship between symptoms and disability in adolescents with IBS, such that the relationship between symptoms and disability was strongest when perceived competence was low [15]. Adolescents with IBS are also more likely to experience depression and anxiety than are healthy counterparts [3], co-morbidities that may further impact quality of life and exacerbate IBS symptoms. As reviewed below, the roles of stress and psychosocial variables are becoming apparent in current conceptualizations of symptom maintenance. Over time, distressing symptoms may increase because the IBS itself can become a significant source of anxiety and fear of pain [16].

In the proposed study, late adolescence and early adulthood was chosen as the target age range in part because of the significant impact of IBS on school and social life as well as the increased level of independence during this period. In California, adolescents are able to drive at 16 years of age. Given that attendance at a group intervention involves transportation considerations, it is likely that independent access to transportation would increase ease of attending the yoga sessions.

### Pathophysiology of IBS

Complex neurophysiological and psychological pathways underlie the experience of IBS. It is now generally recognized that intimate interconnections exist between the enteric motor system and the central nervous system, termed the brain-gut axis, and that these interconnections are involved in altered perception of visceral events, the hallmark of IBS. Although small intestinal bacterial overgrowth (discussed in detail below) has been implicated in alterations in brain-gut communication, visceral hyperalgesia and gut motility remain the primary "biomarkers" for the condition [17].

Visceral hyperalgesia is a consistent physiologic finding in a majority of patients with IBS [18] and is a leading explanatory hypothesis [19]. Sensitivity of visceral afferent pathways or central amplification of visceral input is thought to be related to enhanced perception of visceral stimuli [20,21]. This altered perception of visceral stimuli may translate as hypervigilance, visceral hypersensitivity, and increased autonomic arousal [22]. Under the brain-gut model, enhanced responsiveness of central stress circuits is related to psychosocial and visceral stressors, leading to IBS symptoms. The model describes a relationship between altered cognitive processes, such as anxiety and hypervigilance, and increased ANS arousal, leading to increased visceral sensitivity and IBS symptoms, which can in turn feed back into further perceived stress and symptoms [16,23]. Support for the role of psychological processes in symptom maintenance is derived from studies showing that stressful life events exacerbate IBS symptoms [24]; and mild psychological stress increases visceral perception in IBS patients but not healthy controls [25]. Recently, psychosocial risk markers, including illness behavior, anxiety, sleep problems, and somatic symptoms, were found to be independent predictors of IBS onset in subjects previously free of IBS [26].

ANS dysregulation may at least partially mediate the brain-gut axis. Findings suggest that many IBS patients have altered autonomic responsiveness to visceral stressors, with increased sympathetic and decreased parasympathetic activity, as noted through heart rate variability (HRV) parameters, compared to controls [27]. Thus, cardiac autonomic system activation, demonstrated by HRV parameters, may be a plausible evaluation of autonomic balance related to ANS activity in IBS symptoms [28]. However, IBS characteristics may moderate the role of cardiac vagal tone. ANS imbalances may only be present in 25% of patients and could vary as a function of IBS severity and type (constipation- versus diarrhea-predominant) [29]. As discussed below, yoga is likely to improve functioning in IBS patients through a variety of mechanisms, including improved psychospiritual functioning and reduced visceral sensitivity, in addition to more regulated ANS reactivity for those patients with ANS imbalance. Thus, it is anticipated that yoga will prove beneficial for IBS patients regardless of IBS characteristics and ANS dysregulation.

The hypothalamic-pituitary-adrenal (HPA) axis may also be implicated in symptoms, but research in this regard is equivocal [30,31]. In a recent study comparing cortisol in women with and without IBS, a trend emerged for increased plasma cortisol levels in the IBS group, although cortisol levels were not associated with current IBS symptoms. These results suggest that HPA axis dysregulation may not play a primary role in modulation of symptoms, but rather, indicate generalized upregulation of the central stress system and reduced state of well-being for a range of stress-related disorders [32]. Thus, cortisol will be included in the study as a measure of change in stress and not a specific neuro-enteric mechanism.

A role for small intestinal bacterial overgrowth (SIBO) has emerged suggesting that gastrointestinal (GI) infection can induce long-term inflammatory changes in the GI tract that may be relevant for a sub-group of patients with IBS. It has been found that IBS patients have significantly more SIBO than controls [33] and changes in GI function, such as increased GI permeability, have been reported [34], which may at least partially be caused by a genetic predisposition for reduced anti-inflammatory cytokines [35]. However, it has recently been argued that the presence of SIBO in IBS patients is not fundamental to IBS pathophysiology and may be a by-product of an increased use of proton pump inhibitors [36]. Given discrepancies in whether SIBO represents a distinct pathogenesis, and the current standard of using visceral hyperalgesia as a surrogate biomarker, all qualified potential subjects with diagnosed IBS will be included in the present study, and the same outcomes and mediators will be tested regardless of etiology.

The inclusion of a test of visceral sensitivity, the Water Load Task (WLT), is an innovative aspect of the present study. To date, no research has examined biological changes in IBS patients following mind-body interventions. Although visceral hyperalgesia may be a somewhat unreliable biological marker compared to those applicable in other diseases, it has been recommended as an important outcome for IBS clinical trials [17]. The WLT also provides a stimulus to assess change in ANS response following IY.

### Standard medical treatments are limited

Given the chronic nature of IBS, by definition, therapy is generally protracted. It is recommended that the chronic use of drugs be minimized or avoided because of the lifelong nature of IBS and lack of convincing evidence regarding the therapeutic benefit of current IBS drugs [37]. A recent Cochrane review found no clear evidence that fiber supplements, lactose-free diets, or lactobacillus supplements assist with the management of pediatric recurrent abdominal pain (RAP) [38]. Additional considerations exist for young patients. For example, parents appear averse to children using medications such as antidepressants to treat IBS [39].

Mind-body approaches to address IBS symptoms and quality of life are currently of interest [16] and may prove to complement existing medical treatments. Clinical studies have demonstrated the value of hypnotherapy, cognitive-behavioral therapy, and brief psychodynamic therapy. A recent randomized controlled trial (RCT) used four 90-minute relaxation training sessions incorporating progressive muscle relaxation and breathing in a group of 98 adult IBS patients [40]. Compared to the standard treatment controls, the relaxation group evidenced significantly improved IBS symptom severity and quality of life, and reduced medical visits. In a recent RCT with 53 pediatric patients, gut-directed hypnotherapy was found to have long-term efficacy for children with IBS and RAP and was significantly more effective than standard medical treatment in reducing symptoms; 85% of patients treated with hypnotherapy were in clinical remission, defined as at least an 80% decrease in pain, at a one-year follow-up compared to 25% of the standard care group [41]. Despite the promise of mind-body treatments for functional disorders, there is limited research into approaches such as mindfulness and yoga.

### Yoga as treatment for IBS

Yoga is a discipline developed in ancient India, incorporating and uniting principles of posture, breathing, and meditation, thought to bring physiological and psychological benefits to practitioners. Characterized as a science of self-study and the development of awareness through a series of specific *asanas *(body postures), *pranayama *(proscribed breathing patterns), and meditation, yoga has been embraced in modern Western settings as a form of exercise and stress reduction. Currently, yoga programs are relatively low cost, widely available in the form of classes or through home video/book programs, and, when performed properly, have little risk of adverse effects. Nevertheless, there is a dearth of well-designed RCTs on the specific effects of yoga for chronic pain conditions, particularly among children and adolescents.

Reduced pain and disability and increased quality of life have been reported in RCTs of yoga for various conditions in adults, including migraines and headaches [42,43], rheumatoid arthritis [44], osteoarthritis [45] and chronic low back pain [46]. A review of the literature on yoga for cancer [47] concluded that yoga interventions consistently yield modest improvements across a range of outcomes, including psychological (mood, distress, stress) and somatic (e.g., sleep disturbance) symptoms. Other reviews of yoga for pain, disability, and quality of life [48], as well as depression [49] and anxiety [50], have noted that yoga appears to enhance physical and psychological functioning, although much of the literature is limited by methodological problems, including small sample sizes, unclear description of randomization, and lack of control groups.

Only two studies have focused on the use of yoga for IBS. Kuttner and colleagues [51] provided the only trial of yoga for pain conditions in children and adolescents (25 IBS patients aged 11-18 years). However, this limited intervention consisted of a 4-week home-practice of yoga, subsequent to an initial training session. It is unclear to what degree participants actually practiced yoga and to what extent they adhered to the prescribed yoga protocol. Despite these limitations, Kuttner et al. found that the yoga group exhibited significantly improved post-study IBS symptoms and significantly reduced disability, coping, and anxiety relative to wait-list controls. The only other study that examined yoga for IBS found improved parasympathetic reactivity in a small group of 20-50 year-old males undergoing biweekly yoga classes for 8 weeks compared with an intervention group administered loperamide (2-6 mg/day) [52]. One limitation was the lack of a non-intervention control group. Before novel therapies can be compared to current treatments, it is recommended that efficacy first be established in comparison to non-intervention controls [53]. Both the medication and yoga groups improved equally on decreasing bowel movements and anxiety, indicating yoga may be similar to standard pharmacological intervention. The study also included autonomic responses to a cold pressor task and found no differences between the medication and yoga groups, although neither behavioral nor self-report pain responses to the task were assessed. The yoga group, however, showed significantly enhanced parasympathetic reactivity, as measured by HRV, when compared to controls at the end of the second month of yoga, indicating a decrease in stress response. However, the study was small (n = 9 for the yoga group), studied only male patients even though the majority of adult IBS patients are female, only included diarrhea-predominant IBS, and employed abbreviated health and functioning assessments. Although limited, the findings suggest that yoga may be at least as effective as medication in alleviating IBS-related diarrhea and anxiety, with additional stress-reduction benefits.

The present study will address a number of the limitations of previous studies. Primarily, the current sample will be sufficiently large to detect treatment effects (see Power analyses below) and will involve an RCT with an appropriate control group for this stage of the clinical trial process. The selection of a wait-list control condition was based upon consideration of the literature and a lack of consensus regarding an acceptable attention control condition to compare to yoga. In this preliminary study, the control group will involve patients receiving care as usual for the length of the yoga intervention. Symptoms, physical activity, and lifestyle changes will be assessed weekly in both the yoga and control groups. While the control group will not account for the social, attention, or expectation aspects of the IYP, a usual care control group is appropriate at this stage of the research when examining a novel treatment in an untested population. If the IYP is found to be superior to the wait-list group in this exploratory study, a future R01 will incorporate a control condition that accounts for the social, attention, and expectation effects of a group-based intervention.

**Figure 1:**
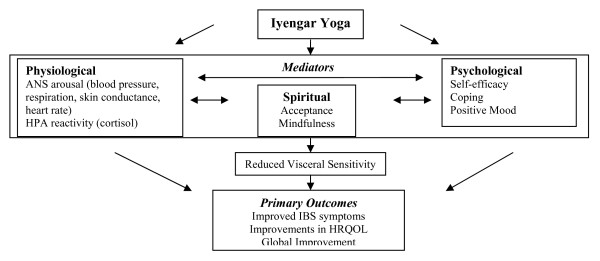
**Conceptual model of Iyengar yoga for IBS**.

Other strengths of the study include the testing of psychophysiological mechanisms of action based on our published model of yoga (see figure 1) [54]. In addition, this investigation unlike previous studies will take developmental issues into account. This study will be limited to later adolescence and early young adulthood. In order to enhance scientific generalizability and the actual practice of yoga, it is important to target an age-range that represents similar challenges. For example, older IBS patients will likely have differences in motility, flexibility, and confidence compared to a younger group. A number of reasons underlie the focus on 14-26 year-olds. First, this group is likely to experience similar developmental challenges, including adjustments in a sense of self, family, schooling, and peers [55]. The primary stressors for most young people of this age-range relate to academic and social performance and increasing levels of autonomy. Second, the level of independence during this period will increase retention of subjects. In California, adolescents are able to drive at 16 years of age. Given that attendance will involve transportation considerations, it is likely that independent access to transportation will increase ease of attending the yoga sessions. Third, the target age range reflects the peak in IBS prevalence that remains relatively consistent across adulthood. During high school, IBS rates increase to adult levels of 15%, from around 8% earlier in childhood [3]. This suggests a developmental shift in the illness around adolescence. It is therefore likely that the IBS symptoms experienced by older adolescents will be comparable to those experienced by college-aged young adults. Overall, it is anticipated that the IYP will have similar benefits for adolescents and young adults across the age group studied. Despite the likelihood that IY will prove useful for both adolescents and young adults, consideration will be given to sub-groups within the age-range. It is possible that a 14-26 year-old range crosses important developmental challenges (e.g., entering college), and results will therefore be examined separately for a 14-18 year-old adolescent sub-group and a 19-26 year-old young-adult sub-group. Thus, feasibility, efficacy, and safety will be examined separately for adolescents and young adults, and only if the sub-groups respond similarly to the intervention will final analyses collapse the groups. Such a developmentally-sensitive approach has not been used in yoga research to date.

Unlike most other studies that have failed to specify the yoga tradition utilized, a planned, standardized intervention from the IY tradition will be employed. IY has been beneficial in treating a range of chronic medical conditions [48] and is likely to be particularly suitable for IBS patients. Sequences are tailored for specific conditions, teachers have extensive training in anatomy, physiology, and safety, and the use of props allows poses to be held without strain or fear of injury. The emphasis on alignment in the IY tradition is unique, and it is believed that maintaining poses with effort on understanding alignment strengthens muscles, organs, and joints, and develops mindfulness of poor posture or gripping of tension in the body [56]. IY is one of a few forms of systemized yoga that involves extensive teacher training. Teacher training occurs in one of 16 Iyengar yoga associations around the world. Under the guidance of Mr. Iyengar, the associations have devised teacher training courses with detailed guidelines on requirements for enrolling, the training syllabus, stages of assessment, awarding certificates, and maintaining certification. Teachers study for at least seven years before being certified to work with students who have therapeutic needs. Tests for certification include teaching, performance of poses and breathing techniques, anatomy, and therapeutic sequences. The detailed sequences of postures tailored for specific health conditions and the rigorous teacher training program suggest IY is particularly suitable for use in clinical trials.

### Biopsychosocial model of chronic pain

The conceptual model for the present study is built within the biopsychosocial model of chronic pain [57]. The frequent co-occurrence of affective disorders, other functional GI disorders, and non-GI complaints suggests that the maintenance and treatment of IBS can best be understood using a biopsychosocial model [16]. It is likely that biological factors such as genetics and visceral hypersensitivity along with psychological and lifestyle factors all contribute towards IBS-related pain and functioning. Twin studies have shown that while genetics play an important role in IBS symptoms, environmental factors such as social learning contribute an equal or even greater etiological influence [58]. Thus, an integrative treatment strategy for patients with IBS is warranted. IY represents a non-pharmacological intervention that can be integrated into a conventional treatment plan. Within the conceptual model below, IY is viewed as impacting IBS symptoms and pain through physical, psychological, and spiritual mediators. Such examination of mediators has been deemed critical, both for understanding treatment components as well as to further our knowledge of functional disorders [59]. Derived from the biopsychosocial model of pain and yoga philosophy, Figure 1 depicts a conceptual model of how IY may improve IBS pain, functioning, and stress. The model offers a guiding heuristic for conceptualizing the benefits of IY for IBS; the model does not depict all possible pathways across domains. The outcomes in the model represent the study's primary outcomes, and the physiological, psychological, and spiritual mechanisms represent the exploratory mediators to be tested.

### Physiological mechanisms

It is thought that yoga quiets the body as well as the mind through attention and vascular and muscular relaxation [60]. Yoga has been associated with a number of physiological benefits. Research linking yoga with ANS functioning has found improved physiologic responses, including reduced diastolic and systolic blood pressure, decreased heart rate, and increased heart rate variability, indicative of healthy functioning [61-64]. IY appears to be especially involved in enhancement of cardiac parasympathetic system modulation, consistent with a reduction in stress response pathophysiology [64]. It is possible that breath awareness during yoga produces a high level of autonomic control. For example, after a month of yoga practice, a group of 12 healthy participants had a lower resting heart rate after the intervention compared to a group of 12 controls; individuals in the yoga group were also able to significantly lower their heart rates when given instruction to do so [65]. It is unknown whether similar autonomic enhancement will be evident in young IBS patients; however, biofeedback, which incorporates autonomic control, has been used successfully with children as young as 8 years of age [66]. Yoga may also impact the endocrine system and HPA reactivity. It is believed that *asana *practice massages the internal organs, resulting in enhanced blood circulation, glandular functioning, and ultimately balancing of hormone production [60]. A number of studies have found changes in cortisol, a measure of stress response system activation, following yoga [67-69]. The physiological benefits of yoga may prove beneficial to many IBS patients, particularly those with ANS and HPA axis dysregulation. Accordingly, cortisol, ANS arousal, and visceral sensitivity will be measured before and after the intervention and compared to the standard care controls.

### Psychological mechanisms

Yoga has a number of positive effects on psychological functioning that have been reported in healthy, pain, and stress groups and for a wide variety of age groups. Two recent reviews examining yoga for depression and anxiety underscored the promise of yoga for elevating mood. Out of five RCTs of yoga for depression, beneficial effects were reported in four [49]. All eight randomized studies of yoga for clinical anxiety disorders reported a reduction in symptoms following yoga [50]. For example, Woolery et al. [67] reported reduced depression and anxiety after a 5-week IYP in depressed young adults. Similarly, Shapiro [70] found that a 20-week IYP led to reductions in depression, anger, anxiety, and neurotic symptoms in 17 patients with major depression (aged 20-71 years). Other positive psychological effects include coping and self-efficacy. Adolescents with IBS were found to have reduced anxiety and less emotion-focused coping following four weeks of home-practice yoga [51]. Persisting in a yoga class promotes a sense of accomplishment that is consistent with the development of self-efficacy. Given the previous role of competence in the link between adolescent IBS patients' symptoms and disability [15], it is likely that a sense of mastery achieved through yoga will positively impact symptoms. Self-efficacy, effective coping, and positive psychological functioning, in turn, are related to better physical functioning in patients experiencing chronic pain and illness [71]. Thus, it is anticipated that yoga will enhance patients' coping, psychological functioning, and self-efficacy, all of which are expected to mediate the hypothesized improvement in clinical symptoms following the IYP.

### Spiritual mechanisms

Related to the psychological benefits of yoga are the spiritual underpinnings of yoga. Spirituality and well-being appear to be connected, particularly in those experiencing chronic illnesses [72]. Two aspects of spirituality that IY is likely to impact are mindfulness and acceptance, both of which will be tested as mediators in the present study. IY is associated with a mastery of life challenges, which may extend to dealing with chronic illness and pain [73]. As noted by BKS Iyengar, "The pain is there as a teacher... We must try not to run from pain but to move through it and beyond it" [74]. It is anticipated that the IYP will enhance patients' compassionate understanding, an Eastern concept that in this context can be conceptualized as compassion towards oneself or acceptance (i.e., acceptance of one's health condition). Such acceptance or understanding of one's state appears to be important for those with chronic pain [75]. IY is also associated with mindful awareness, a concept that has been integrated in empirically-validated approaches including cognitive-behavioral therapy [76]. Mindfulness is an openness or receptive awareness to what is occurring in the present. Mindful activity has been found to improve mood and stress [77] likely through strengthening attention [60]. Use and control of attention can be turned towards minimizing stress, disability, and pain. It has been theorized that mindfulness may be especially effective in treating functional disorders such as IBS [16]. It is anticipated that IY will lead to an increase in mindfulness and acceptance which will in turn improve functioning among young IBS patients.

## Aims

The purpose of the proposal is two-fold: 1) to examine the effects of an IYP on IBS symptoms, global improvement, and quality of life in adolescents and young adults aged 14-26; and 2) to explore the mediating roles of physiological and psychospiritual processes through which IY is likely to impact outcomes.

The specific aims of the proposed project are:

1. To determine the safety, feasibility, and acceptability of the IYP in IBS patients aged 14-26 years.

2. To test for changes in clinical symptoms (primary outcomes), including self-reported IBS symptoms, global improvement, and health-related quality of life (HRQOL) immediately following a standardized IYP and at 2-month follow-up compared to usual care wait-list controls.

3. To assess changes in visceral sensitivity (assessed via a standardized laboratory visceral sensitivity task), disability and psychospiritual functioning (i.e., catastrophizing, mood, self-efficacy, acceptance, and mindfulness) (secondary outcomes) following the IYP compared to controls.

4. To explore potential mediators of clinical outcomes following the IYP, including psychospiritual functioning, visceral sensitivity, and ANS and HPA activity.

The hypotheses to be tested are:

1. The IYP will be safe, acceptable and feasible: at least 80% of subjects will complete the IYP.

2. Following the IYP, participants will demonstrate significantly greater improvement on the primary clinical outcomes of IBS symptoms, global improvement, and HRQOL relative to wait-list controls; gains will also be evident at the 2-month follow-up.

3. Following the IYP, participants will demonstrate significantly reduced visceral sensitivity and disability, and improved psychospiritual functioning, including coping, mood, self-efficacy, acceptance, and mindfulness (secondary outcomes) relative to wait-list controls.

4. Exploratory hypothesis: The impact of the IYP on the primary clinical outcomes will be mediated by improved psychospiritual functioning, reduced visceral sensitivity, and improvements in ANS and HPA dysregulation.

## Research design and methods

### Design

**Figure 2:**
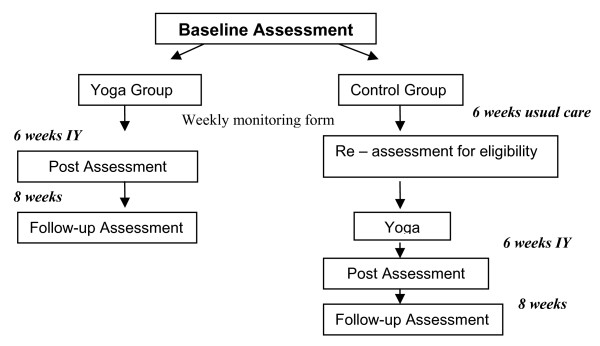
**Flowchart of study design**.

A randomized control design will be used to examine the impact of IY for youth aged 14-26 years with IBS on the following outcomes: IBS symptoms, global improvement, HRQOL, and laboratory pain sensitivity. The study design is depicted in Figure 2. After baseline assessment, including standardized questionnaires and the laboratory visceral task (including ANS/psychophysiological measurements and cortisol collection), participants will be randomly assigned to the IY program or the wait-list control group. It is anticipated that randomization will ensure equal distribution of males and females, adolescents and young adults, and symptom severity across groups. Baseline assessments will be completed in order to ensure that the yoga and control groups are comparable, and to test for pre/post changes in the yoga group.

#### Randomization

A research assistant who is not otherwise involved in the yoga study, and who has not met or had previous contact with any of the subjects, will use the Research Randomizer program as a means to generate random numbers (either 0 or 1) for patient assignment to the intervention or wait-list group. The program uses a JavaScript random number generator to produce customized sets of random numbers, which are then downloaded in an Excel database. Patients who have been screened and have entered the study will be listed in the recruitment database. This list of participants will be aligned with the output of random numbers. Patients with a number less than 0.5 will be assigned to the intervention group and those with a number greater than 0.5 will be assigned to the wait-list group. Consistent with the need to ensure allocation concealment [78], the randomization results will be kept in a secure, locked, password-protected Excel database that only the research assistant has access to.

Female participants will provide information on their last menstrual period during the baseline questionnaire and laboratory session, and where possible, their post-intervention questionnaire and laboratory session will be scheduled to take place during the same cycle phase as their baseline session (e.g., follicular vs. luteal phase). Pain may vary for women along with their menstrual cycle, both for laboratory pain tasks [79] and for general IBS symptoms [80]. It is therefore important to ensure that any change experienced by female patients after the IYP is not the result of completing the laboratory task during a more/less sensitive phase of their cycle.

Those initially assigned to IY will then engage in the 6-week biweekly program (each session will last 1.5 hours; total duration = 18 hours), while those assigned to the control group will receive care as usual. All participants will repeat the baseline assessments, including the sensitivity task and psychophysiological assessment and cortisol collection, immediately following the total treatment/control period. Questionnaires regarding symptoms will also be e-mailed at a 2-month post-treatment follow-up. Both the yoga and control groups will receive a weekly monitoring form to assess symptoms, physical activity, and lifestyle changes (and to ensure that the control group does not participate in any mind/body interventions that may impact findings). The weekly monitoring form will be administered by phone or e-mail to both groups at the end of each week. After the 6 weeks, controls will be re-assessed to ensure that they still meet study eligibility criteria; those who continue to meet criteria will take part in the IYP. Following IY, participants will repeat the baseline assessments at post-treatment and complete the 2-month post-treatment follow-up questionnaires.

According to the Power/Sample Size calculations outlined below, 30 patients in each of the intervention and control groups will be recruited (total n = 60). To accommodate this number, 4 IY groups and 4 initial control groups will be conducted, with approximately 8 patients per group.

### Study participants

#### Enrollment criteria

Male and female youth will be eligible for the study if they meet the following criteria:

• Age 14-26 years.

• Diagnosis of IBS, using ROME III pediatric criteria for patients aged 14-17 years, and ROME III adult criteria for 18-26 year-olds.

• Able and willing to give written informed assent or consent and comply with the requirements of the study protocol.

• Ability to speak and understand English.

Every step will be taken to ensure that equal numbers of participants make up the sub-groups, including equal numbers of young men and women and young people across the entire age group studied.

#### Exclusion criteria

Patients will be excluded from the study based on the following criteria:

• History of drug, alcohol, or chemical abuse within 6 months prior to screening.

• Any other injury, disease, metabolic dysfunction, physical examination finding, or clinical laboratory finding giving reasonable suspicion that it might affect the interpretation of the results or render the patient at high risk from treatment complications.

• Inability to comply with study and follow-up procedures.

• Currently pregnant.

• Previous practice of IY within the past three months.

• Inability to speak and understand English.

• Plan to begin a new treatment within 2 weeks of the IYP.

Because a number of the outcome measures have been validated in English only and the IY protocol is only in English, for this initial study only those patients able to speak or understand English will be included. However, in future large-scale trials, Spanish speaking individuals will be included and we will translate and back-translate the questionnaires into Spanish if no Spanish versions exist, as well as develop yoga protocols that are in Spanish, and employ Spanish speaking IY teachers. We will ask participants whether they are pregnant at the time of study entry; however, the IY classes will not contain poses that are harmful to pregnant women. Substance/alcohol abuse, as well as practice of yoga, will be assessed via self-report. Although subjects will not be excluded if they begin new treatments during the IYP, those subjects who are planning on introducing new treatment 2 weeks or less before the IYP commences will be excluded. These patients will be offered participation in the next round of IY classes.

#### Recruitment

Participants will be recruited from the pediatric gastroenterology clinic and the UCLA Center for Neurobiology of Stress (CNS) in the Division of Nutrition and Digestive Disease at the David Geffen School of Medicine at UCLA, as well as from the UCLA Pediatric Pain Program (PPP) clinic, which sees patients up to 22 years of age. Recruitment will also occur at various medical offices specializing in digestive disturbances. Participants will also be recruited through community resources, such as library and other public notice boards, as well as websites of various IBS support groups and Craigslist. We will also recruit participants from press releases sent to surrounding areas through the UCLA Health Sciences Media Relations department. Figures at the UCLA Division of Digestive Diseases and the PPP suggest that approximately 15 IBS patients per month will be recruited in the appropriate age group. Private practice digestive specialists in Los Angeles who have agreed to refer to the project can refer an additional 18 patients with IBS per month in the targeted age range.

The study administrator will meet with health care providers, staff, and support group leaders to inform them about the project and ask them to distribute and post flyers about the study. The flyers will describe the project and ask interested volunteers to call in to establish their eligibility. Effort will be made to recruit the patient sample to be broadly representative of the general population of IBS patients in the targeted age range. Early epidemiological data examining the racial/ethnic composition of IBS in a Southern California Health Maintenance Organization (HMO) suggest that approximately 82% of patients will be White (non-Hispanic), 8% Hispanic, 3.5% African American and 1% Asian [81]. Adult women are twice as likely to be affected, and sex differences in IBS generally begin in late adolescence [81]. The study is therefore likely to have a greater number of female patients. Recruitment efforts will attempt to reflect the demographic composition of the Greater Los Angeles area which is estimated to be approximately 75% White, 13% Asian, 10% African-American, 1% American Indian/Alaska Native, and 1% Native Hawaiian/Other Pacific Islander.

### Intervention

#### Iyengar yoga program

The poses to be included in this intervention are based on the teachings of BKS Iyengar. They will include supine poses, passive backbends, standing poses, and supported inversions (e.g., Down Dog on Ropes with chair; Rope headstand; Chair backbend on two chairs; Chair shoulder stand; Chair Halasana; Setubandha Sravangasana on Bench; Viparita Karani; Supta Baddhakonasana; and Supta Pada Ghustasana). All will be done with props as needed. Young IBS patients will be taught an IY practice that is specifically designed to address their abdominal issues. The yoga poses will be done with the aid of props (blocks, bolsters, chairs, straps, and blankets) to reduce tension and strain, promote circulation, and reduce apprehension of pain. The practice will help bring awareness to habitual patterns of holding tension, as well as increase range of motion and strength. Students will learn to tolerate increased sensation without gripping and creating stress as they develop deeper states of relaxation and calm. The intervention will be conducted by an experienced IY teacher. At the end of the 6-week IYP, participants who ask for referrals to IY studios/teachers in the community will be given a list of referrals. Participants will be provided with props during week 3 to begin an informal home practice, if they wish. All home practice as well as post-IY classes will be monitored during the weekly monitoring form and at follow-up.

#### Dose

The proposed IYP will be administered twice a week for 1.5 hours duration per session for 6 weeks. The length of the IYP, class duration, and number of sessions was based on our previous research using IY for young populations. We found significant effects after a 5-week biweekly (90-minute sessions) IYP for depression in young adults. However, these participants did not have clinical levels of depression or a chronic condition, and a longer intervention is appropriate for the present patient population. Most recently, we conducted a pilot study of a 6-week biweekly (90-minute classes) IYP for young adults with RA [82] and found significant reductions in pain and symptoms. It was therefore deemed suitable to strive for the maximum benefit in the shortest time likely to achieve benefit (6 weeks). The weekly monitoring form (described below) will track symptoms over the study period and help to further inform studies about appropriate dose for young people.

#### Yoga adherence monitoring board

A board will be created with the purpose of monitoring instructor adherence to the study manual. The board will consist of two senior IY teachers, who will conduct ratings for teacher adherence by reviewing notes from the classes. A research assistant will attend a random selection of 25% of classes to note poses completed, use of props, and any modifications or difficulties experienced. Using a checklist, the board will rate the extent of adherence to the yoga manual.

### Laboratory pain task

The following task designed to measure visceral sensitivity will be administered in the PPP laboratory both at baseline and then post-intervention following the IY intervention/control groups.

#### Water load task

(WLT) to assess visceral sensitivity. Visceral sensitivity tasks are designed to challenge patients for sensitivity to visceral sensations. Walker and colleagues [83-85] developed the Water Load Pain task, which creates visceral sensations similar to pain episodes in children with abdominal pain. Water is administered orally as a single trial of visceral sensation of abdominal discomfort. This task correlates with the threshold for pain/discomfort measured using an electronic barostat and has been shown to be safe in healthy and clinical samples of adults and children; and it is far less intrusive than other measures of visceral sensitivity, such as rectal distension [86,87]. After a two-hour fast, subjects will be asked to drink non-caloric, non-carbonated filtered water at room temperature in as large a quantity as possible until s/he feels completely full or reaches a limit of intolerability with an uninformed ceiling of 15 minutes. Subjects will be informed that this is 'not a race.' Subjects will be blinded as to the total volume ingested. The time (seconds) elapsed from the beginning of water ingestion until the subject stops drinking will be recorded.

#### Measures

*To assess the outcomes and mediators depicted in the conceptual model (Figure*1)

## Primary outcomes

### Questionnaires at baseline, post-treatment, and follow-up

**1**. ***IBS symptoms ***will be measured using the ***IBS severity scoring system (IBS-SSS) ***[88]. The scale measures the sum of the participant's evaluation on a 100-point scale of each of five items: severity of abdominal pain, frequency of abdominal pain, severity of abdominal distension, dissatisfaction with bowel habits, and interference with quality of life. The IBS-SSS has acceptable reliability and validity and is recommended for use in outcome studies [89]. It takes approximately 5 minutes to complete. Those younger than 18 will also receive the abdominal symptoms sub-scale of the Child Somatization Inventory (CSI). The CSI [90-93] measures perceptions of nonspecific somatic symptoms. Individuals rate how much they are bothered by each of 24 symptoms (e.g., headaches) during the last 2 weeks using a 5-point scale (*not at all *to *a whole lot*). It takes less than 5 minutes to complete.

**2. *IBS pain ***will be measured with the ***Abdominal Pain Index ***comprising five items assessing the frequency, duration and intensity of pain episodes over the previous 2 weeks. Good reliability and validity have been reported [94].

**3**. ***Global Improvement ***will be assessed with the ***Global Improvement Scale **(**GIS**)*. This scale asks participants "Compared to the way you felt before you entered the study, have your IBS symptoms over the past 7 days been from (1) = substantially worse, to (7) = substantially improved [95]". Global improvement is noted for patients endorsing moderately or substantially improved status. The scale shows adequate reliability and validity [95]. Global improvement, in addition to symptom severity, is recommended as a primary endpoint in IBS therapy trials [96]. The scale takes less than 1 minute to complete.

**4**. ***Health Related Quality of Life ***will be assessed with the ***Health Related Quality of Life - Short Form-36 (SF-36) ***[97] a generic core HRQOL measure yielding an 8-scale profile of functional health and well-being. The SF-36 performs well in an absolute sense and comparatively better than other HRQOL measures in terms of reliability, validity, and lightness of respondent/administrative burden [98]. It can be completed in 5-10 minutes and has been validated in children as young as 10 years of age [99].

#### Secondary outcomes

**1. *Functional Disability Index (FDI) ***[92] is a 15-item measure assessing perceived difficulty in physical and psychosocial functioning in the past 2 weeks due to physical health. Items are rated on a 5-point scale (*no trouble *to *impossible)*. It takes less than 5 minutes to complete.

**2**. ***Visceral Sensitivity Index (VSI) ***is a reliable, valid measure of gastrointestinal symptom-specific anxiety [100]. It has 15 items assessing anxiety related to gastrointestinal sensations that are rated on a 6- point scale from *strongly agree *to *strongly disagree *and takes less than 5 minutes to complete.

3. *Laboratory assessment at baseline and post-treatment*

**Visceral pain sensitivity **will be assessed via the **water load task**, a measure of visceral pain. Laboratory pain tasks provide a standardized, controlled way of assessing pain sensitivity. The laboratory procedure will take approximately 30 minutes.

##### Self-report and behavioral pain sensitivity

1. Pain Intensity: Self-report ratings of pain intensity will be recorded on a 10 cm vertical visual analog scale (VAS) used to represent a continuum from *no pain at all *to *worst pain imaginable*. The VAS has been widely used as a valid and reliable measure of pain intensity with children [101] in experimental and clinical pain studies.

2. Pain Tolerance: Maximal water intake (mL), adjusted for BMI, will be recorded. Tolerance represents a behavioral measure of pain response.

3. Pain Discomfort: Subjects will be asked to rate their discomfort using the VAS to represent a continuum from *no discomfort at all *to *worst discomfort imaginable*. The measure will assess the affective component of the pain. Participants will be asked 'how much does your stomach feel bad or uncomfortable?'

4. Anticipatory Anxiety: Before each pain task, subjects will use the vertical VAS automated slider to rate how anxious or nervous they are about performing the upcoming task, in response to the question 'how nervous, afraid or worried are you about the task?'

5. GI and Non-GI Symptoms: The Symptom Emotion Report is a 21-item measure developed by Dr. Lynn Walker's lab that measures GI (e.g., nausea/upset stomach) and non-GI (e.g., tired, upset) symptoms experienced immediately before and after the WLT on a 5-point numerical scale of 0 (*Not At All*) to 4 (*A Whole Lot*). Williams et al. [102] found that GI symptoms reported during abdominal pain episodes significantly predicted GI symptoms experienced following the WLT.

#### Psychospiritual functioning

**1. *Self-efficacy ***will be assessed with the ***Pain Self-Efficacy Questionnaire (PSEQ) ***[103], a brief self-report of self-efficacy for functioning despite chronic pain. The scale consists of 10 items, takes 2 minutes to complete, and asks about functioning when in pain; functioning domains include activities related to school, friends, family and work (including household chores). Acceptable reliability and validity have been reported [103].

**2. *Pain Catastrophizing ***will be assessed with the ***Pain Catastrophizing Scale (PCS) ***[104] a 13-item self-report instrument based on the catastrophizing subscale of the Coping Strategies Questionnaire [105]. Respondents select one of five response options (*not at all *to *all the time) *indicating the degree to which they experience each item (i.e. "When I'm in pain, it's terrible and I think that it is never going to get any better"). This measure takes less than 5 minutes to complete.

**3**. ***Mood ***will be evaluated using the ***Brief Symptom Inventory 18 (BSI-18) ***[106]. Respondents rate how often they have experienced anxiety, somatization, and depressive symptoms within the past 7 days on a 5-point scale ranging from *not at all *to *extremely*. The BSI-18 has shown adequate to good internal consistency (α range = .74 - .89) and validity [106]. The BSI-18 requires a 6^th ^grade reading ability and takes approximately 5 minutes to complete. Children under 18 years will be administered the Revised Child Anxiety and Depression Scale (RCADS) [RCADS; [107,108]], a 47-item adaptation of the Spence Children's Anxiety Scale (SCAS) [SCAS; [109,110]]. This measure assesses children's report of symptoms corresponding to selected DSM-IV anxiety disorders and depression. This measure takes 10 minutes to complete.

**4. **The ***Chronic Pain Acceptance Questionnaire (CPAQ) ***[111] is a 20-item measure to assess acceptance of chronic pain. The CPAQ has two subscales: Activity Engagement assessing patients' participation in activities regardless of pain and Pain Willingness, assessing absence of attempts to control or avoid pain. The CPAQ has acceptable reliability (*α *= .78-.82) and validity [111]. It takes approximately 5-10 minutes to complete and is suitable for older adolescents as young as 16 years of age.

**5. *Mindfulness ***will be assessed using the ***Mindfulness Attention Awareness Scale (MAAS) ***[76], a 15-item measure rated on a 6-point Likert scale. Internal consistency is high (α = .82) and the scale has acceptable convergent and discriminant validity [76]. The scale has been used with adolescents and young adults [112] and was initially developed with participants aged 17 years and above. It takes approximately 5 minutes to complete.

### Exploratory mediators

#### Laboratory physiological responses at baseline and post-treatment

##### Physiological responses

Noxious stimuli such as pain or emotional distress are known to activate the neuro-hormonal stress response [113]. The stress response is modulated primarily by corticotropin releasing hormone (CRH) and the autonomic nervous system. Pain has been shown to activate the hypothalamus (hormonal response) and the periaqueductal gray (autonomic nervous center) [114]. The overall physiologic expression of the stress response is manifested from a balance between hormonal influences, sympathetic tone, and parasympathetic tone. The acute stress response is characterized by increase in heart rate, blood pressure, respiratory rate, skin conductance, and cortisol.

##### Autonomic responses

Autonomic arousal will be determined from measures of skin conductance, heart period, respirations, and blood pressure recorded continuously during the lab sessions using a BIOPAC MP150 recording system (BIOPAC, Inc., Santa Barbara CA). Briefly, skin conductance (SC), which reflects peripheral sympathetic activity, is recorded using Ag/AgCl electrodes secured to the volar surface of the first and third fingers of the non-dominant hand. Heart period (HR) is recorded using a two lead ECG from electrodes attached to the jugular notch and over a lower rib. HR and SC are sampled at 1000 hz and analysis of skin conductance level, skin conductance responses, average HR, and heart rate response during each task is computed using BIOPAC software (for peak detection and averages) and specialized software developed in our laboratories over the past 10 years. Blood pressure is recorded using a noninvasive system supplied by BIOPAC (NIBP100A), which records radial artery waveform from a non-occluding wrist cuff. Using a patented algorithm the NIBP100A system calculates accurate systolic, diastolic, and mean pressures and has been validated against arterial recordings. Respiration is recorded using an abdominal belt and used to check for unusual breathing patterns that might impact interpretation of the other measures. Heart rate variability (HRV) is determined from the interbeat intervals using specialized spectral analysis software developed for this purpose [115,116], which produces separate area and peak power measurements for the low frequency (parasympathetic and sympathetic influences) and high frequency (parasympathetic or vagal) components of heart period. Heart rate variability will be assessed by including a measure of respiration (to calculate respiratory sinus arrhythmia). Boyce and colleagues [117] have established the reliability and validity of this system for physiological recording in children as young as 3. Cardiac vagal tone is also estimated using a software filter to detect respiratory sinus arrhythmia (RSA, Delta-Biometrics, Bethesda, MD). RSA is the normal variation in heart rate occurring in synchrony with the inspiratory and expiratory phases of the respiratory cycle. ANS variables will include resting and maximum change values during each task for: blood pressure, skin conductance, high frequency HRV (vagal tone), and sympathetic balance (HF/LF).

##### Cortisol

Salivary cortisol, a neuroendocrine marker of HPA axis reactivity has long been used in the study of the psychobiology of stress [118]. Salivary cortisol will be used as a physiological evaluation of general stress, and whether patients' stress response changes as a result of the IYP. Collection of cortisol through saliva is a non-invasive way of measuring HPA axis activity and is correlated with unbound serum levels [119]. Fifteen minutes prior to the first collection, participants will be asked to rinse their mouth with water to prevent contamination of sample by food debris. Subjects will also be instructed not to eat or drink 1 hour prior to the session to minimize sample contamination. Participants will be instructed to imagine smelling and eating their favorite food and to begin to allow the saliva to pool at the bottom of their mouths. Saliva samples will be collected with small straws placed in the mouth so that the saliva can easily be collected into a cryovial. We will aim to collect 1.5 mL of saliva at each collection from each participant. Saliva is then frozen and sent to the UCLA CNS Endocrine Core laboratory for quantification. Salivary cortisol will be collected before and after each laboratory session as well as the morning of the laboratory assessments. Each laboratory session will involve three samples: immediately upon arrival (initial baseline measure); directly after completing the questionnaires (second baseline measure); and 20 minutes after the end of the visceral sensitivity task (maximal stress response). A meta-analysis of acute psychological stress reveals that the overall peak cortisol response occurs 21-40 minutes from onset of an acute stressor level [120]. One of the most prominent features in studies of adrenocortical responses under physical and psychological stimulation is the large variation in both baseline levels and in response magnitude between subjects. We intend to assess salivary cortisol twice before the pain tasks to control for individual differences at baseline and to capture the possible effects of anticipatory stress on cortisol elevation. These multiple assessments should reflect the overall magnitude of cortisol changes [121].

### Additional measures

**1**. An **expectation **question will be administered after assignment and before treatment to assess whether treatment expectation impacts outcomes. Subjects will be asked to rate on a scale of 0 to 10 'How much do you think the treatment will benefit you or improve your IBS symptoms?'

**2**. The **weekly monitoring form **will include items related to mood, IBS pain, symptoms and functioning and use of any new interventions. The form will be administered by telephone or e-mail at the end of each week to both groups. The weekly monitoring form is an important tool in the study design, given that IBS is a condition without clear biomarkers to assess illness severity and progression, self-reported pain, symptom and functioning tools will form the basis for assessment of appropriate dose. The form will also ensure the control group does not introduce any new mind-body treatments during the intervention.

**3**. Females will receive a question that asks about their last menstrual period (LMP) to control for potential varying pain sensitivity over cycle phase. Self-reported menstrual cycle length is a common, non-invasive clinical marker for reproductive function [122].

Participant Burden: It is anticipated that the laboratory procedure and the standardized questionnaires will take 1.5 hours at baseline, and 1.5 hours post-treatment. The 2-month follow-up is expected to take 40 minutes. The weekly monitoring form will take approximately 8-10 minutes each week.

### Data management

A unique ID will be assigned to each patient for the purpose of protecting anonymity, and no patient identifiers will be included in the dataset. Data from the standardized quantitative measures will be entered into an Excel database then moved into SPSS, which will be used for the statistical analyses. Initial data cleaning and preparation will include evaluation of the distributions, outlier/error detection, logical and range checking and creation of derived variables. Sensitivity analyses will be conducted after imputing for missing data.

### Data analyses

Each stage of data analysis detailed below will be carried out separately for adolescent (14-18 yrs) and young adult (19-26 yrs) sub-groups. If no differences are found, the groups will be combined. It is unlikely that differences will emerge in terms of feasibility, efficacy or safety, but a separate analysis of sub-groups will ensure that findings are generalizable across the ages studied.

Once the data set has been cleaned and examined for skewness, exploratory data analyses will be conducted using univariate methods to describe the variables of interest and bivariate techniques to characterize their interrelationships within our sample. Continuous variables will be summarized over time in each group using means, medians, range, interquartile range and standard deviations. Cross tabulated frequencies will be given for all discrete variables by group and time. Both parametric and non-parametric correlations will be reported. Expectation ratings will be correlated with primary outcomes to determine whether patient expectations were related to post-intervention symptoms.

For **Hypothesis 1**: IY will be safe, acceptable and feasible: more than 80% of subjects will complete the training program, the frequencies of drop-outs for the IYP and control groups will be examined. Attrition rates will be compared between the yoga and control groups; demographics and baseline values of functioning between those who complete the protocol and those who do not will also be compared. If drop-outs are random, then the results may be considered unbiased. If drop-outs are not random, the direction and magnitude of potential biases will be considered.

To test **Hypothesis 2: **Participants in the IY group will demonstrate significantly greater improvement in IBS symptoms, global improvement and HRQOL apparent immediately post-intervention and at the 2- month follow-up. Consistent with recommendations regarding analyses for randomized trials, analysis of covariance (ANCOVA) will be used to test group differences [123]. The baseline scores of the outcome variables will be entered as independent variables. The Next, analyses will assess maintenance of treatment gains during the follow-up period. Changes in IBS symptoms, HRQOL and improvement across baseline, post-treatment and follow-up will be evaluated using ANCOVAS. An intent-to-treat (ITT) analysis on the primary outcome measures will be conducted to confirm findings in treatment completers by including all participants who enrolled in the study, using the last observation carry forward approach. Even if significant differences are noted, consideration will be given to established minimally clinically significant differences on outcome measures between the groups. Thus, in order to be considered a clinically meaningful difference, participants must evidence a decrease in at least 50 points on the IBS-SSS in comparison to controls.

To test **Hypothesis 3: **Participants in the IY group will report significantly reduced visceral sensitivity, disability and improved psychospiritual functioning. The same methods will be used as described above for hypothesis 2. Similar analysis will be conducted to examine changes in visceral sensitivity, disability and psychospiritual functioning, including self-efficacy, coping, mood, acceptance and mindfulness from baseline to post-treatment.

To test **Hypothesis 4: **As depicted in Figure1, Psycho-spiritual-physiological outcomes will be explored as mediators of the association between IY and improved IBS outcomes. Regression analyses will be conducted to determine whether these variables mediate treatment outcome. The procedures outlined in Cohen and Cohen [124] will be used to calculate residualized change scores for IBS symptoms, global improvement, HRQOL and pain sensitivity scores. Multiple regression analysis will then be conducted with IBS symptoms, global improvement, and HRQOL separately; change scores (baseline minus post-treatment) will be regressed onto a set of predictor variables including group (yoga vs. control), the mediator variable of interest (e.g., self-efficacy), and the interaction between the mediator and group. If evidence of mediation is found using the traditional Baron and Kenny approach - the Sobel test - a statistical test for mediation will be conducted to calculate mediation. Finally, exploratory analyses will be conducted from the Weekly Monitoring data.

#### Sample size determination and power analysis

Based on our previous pilot work using Iyengar yoga for patients with rheumatoid arthritis [82], we estimated that yoga was likely to have a medium effect size on the primary outcomes. For power calculations, a freely-available software for power calculations - G*Power 3- was used to estimate the sample size needed for the estimated effect size using a pre/post by group interaction. Pairing a medium effect size with a range of correlation values (.10 to .50) for baseline and follow-up scores resulted in power calculations ranging from 60 to 34 total subjects. These calculations used a Type I error rate of α = .05, and statistical power of 0.80 (the Type II error rate, 1- β). We settled upon the middle value of 47 total subjects. Assuming a 20% attrition rate, it was estimated that 60 total subjects would need to be recruited (30 per group).

### Protection of human subjects

This study was approved by the Medical Institutional Review Board 1 (MIRB1) within the Office of the Human Research Protection Program at UCLA (IRB Number 09-05-070-01). The study will be carried out in compliance with the Helsinki Declaration.

#### Risks to subjects

Yoga is generally regarded as safe and adverse effects are rare, especially within the Iyengar program in which props are used for support and teachers undergo vigorous training for certification. However, a number of case reports have indicated adverse events following yoga, specifically, the presence of subcutaneous emphysema [125] and shortness of breath and chest pain [126]. Nevertheless, these events were in response to intensive *pranayama*, or breathing exercises, with the shortness of breath case experiencing difficulty following *kapalabhati pranayama *involving strong forced breaths which may push the body to physiologic extremes [126]. Such intensive breathing techniques will not be used in this yoga intervention. Great care will be taken in developing the yoga program to ensure that bodily systems are not stressed.

The laboratory pain component of the study presents some potential risks to subjects. During physiological assessments, some subjects may be bothered by arm cuff inflation for blood pressure measurement. However, all participants should have had experience with these procedures from routine medical check-ups. Additionally, some physical discomfort may be experienced during the water load task. However, subjects are in control of how long the task takes--they may stop the procedure at any time and the experimenter will be in the room at all times to provide supervision and support. Also, the experimenter will discontinue the study if distress is observed in the subject whether or not the subject expresses distress. During the procedure, subjects may experience some potentially discomforting anxiety. However, the anxiety will be transitory and is unlikely to exceed levels of fearfulness that subjects experience naturally when unexpectedly encountering mild pain stimuli in their own environment. In past pain studies, we have not encountered any anxiety-related problems. Participants may also feel uncomfortable disclosing personal information about their mood and symptoms on study questionnaires.

### Protection against risk

#### Privacy and confidentiality

Confidentiality will be maintained by the use of unique identification numbers. All data, as well as participant lists, will be kept in locked file cabinets with access restricted to research staff. All data forms will have subject ID only.

#### Safety aspects of the study design and conduct

The major potential risks from the yoga class are strains or injuries. We will minimize these risks by conducting the yoga intervention in the safest manner possible. A progressive series of poses has been developed for this trial; this gradual progression, tailored to the participants, is designed to avoid injury. In addition, props will be used to support participants in more challenging poses. The general safety parameters of the yoga program are: 1) heart rate will not exceed moderate training range (115 BPM) - we will ask participants to check their pulse at the end of each yoga session during a timed one-minute period and to report their heart rate (anyone who remains at a rate of greater than 115 BPM will be interviewed and assessed by a physician to determine if this is a consistent finding or if there may be other isolated reasons for the tachycardia, such as a viral illness); 2) attention will be paid to spinal safety; 3) balance will be carefully monitored (e.g., chairs are used to assist balance as needed). Students will be given modifications of postures using props to accommodate for individual differences and limitations and receive individual attention if postures are too challenging.

To minimize potential risks or discomfort associated with the laboratory tasks, an experimenter will remain in the room for supervision and observation of subject responses. If subjects show signs of acute distress (e.g., tearfulness), the experimenter will stop the experiment and talk with the individual until he/she feels calmed. The patient can end the study participation or continue, depending upon how he/she feels. The team has developed ways of carrying out laboratory pain research with children to minimize threat and to make it an interesting and pleasantly novel experience.

To minimize any potential risks or discomfort associated with study assessments, all assessments will be conducted in a private room. Participants will be encouraged to raise questions or concerns at any time and be reminded that they may withdraw at any time without penalty. In addition, a clinical psychologist will train all research personnel to identify and report any history of abuse or risk for potential self- or other-harm if this information is revealed in responses to questionnaires or during interviews, as required by the study institution's IRB.

### Data and safety monitoring plan

The principal investigator will be responsible for maintaining quality control over the data collection process and for securing its confidentiality; she will oversee the process of data collection and ensure that data is reviewed for completeness and accuracy. Reviewed data will be secured in locked file cabinets in the project offices. During each of the treatment waves, potential threats to the safety and well-being of participants will be carefully monitored. If, for example, there is any adverse change in the medical condition of participants, the study physician will be immediately consulted and the participant's physician contacted to make arrangements for treatment, if necessary.

The proposed project is a single site study with low complexity and minimal toxicity risk. Nevertheless, a safety monitoring committee is appropriate for oversight of the research that includes safety officers who are IBS experts.

#### Monitoring process

Study performance and safety monitoring will be conducted. The list that follows shows the major items that will be included in the monitoring reports. The safety committee will receive bi-monthly summaries containing information on each item in the list, presented in a blinded manner. A trained research assistant will be in charge of preparing reports for the safety committee. In case of safety concerns (primarily about side effects), requests by the committee for unblinded information will be made to the study physican who will provide the necessary information.

#### Content of safety reports

The following performance and safety characteristics will be tabulated monthly and reported to the safety committee bi-monthly. Information on the date, time and nature of any adverse events will be reported separately for the yoga intervention and the visceral sensitivity task.

Performance summary:

1. Recruitment rates, including number of subjects who are screened, percent eligible at each stage of screening, reasons for non-eligibility, baseline visit rates, and randomization figures

2. Demographics of screened and enrolled participants by treatment group

3. Adherence of participants to study treatment (i.e., class attendance)

4. Percent of participants evaluated at each follow-up point and reasons for losses to follow-up

Safety summary:

Iyengar yoga intervention

1. Worsening of symptoms, as documented by the weekly monitoring form

2. Possible side effects, including any cardiovascular event during class

3. Injuries occurring in class or during home practice

4. Any other unrelated, possibly related, and related adverse events by subject

5. Attribution of adverse event to the study intervention, determination of whether the adverse event was expected or unexpected, and study adjustments as necessary

Laboratory visceral sensitivity task

1. Possible side effects, including any cardiovascular event

2. New pain that persists by the end of the lab session

3. Any other unrelated, possibly related, and related adverse events by subject

4. Attribution of adverse event to the study intervention, determination of whether the adverse event was expected or unexpected, and study adjustments as necessary

### Significance

Only one published study has examined the effects of IY as a therapy for young people with chronic pain [51], and methodological limitations preclude strong conclusions from this prior work. The proposed study is innovative as it will be the first controlled investigation of a group-based, standardized intervention of yoga for young people with IBS. IY is a tradition of yoga that is unique in its emphasis on precise anatomical alignment, use of supportive props, sequences of *asanas *(postures) tailored for specific conditions, and a highly systematized and rigorous teacher training system. In combining these elements, IY is likely to be a safe, standardized, and reliable treatment suitable for clinical research. Few studies have included young patients, or a control group, as proposed here. Additional innovations include the testing of psychospiritual and physiological mechanisms of action within a biopsychosocial model, the use of an experimental paradigm to examine patients' visceral pain sensitivity following yoga, as well as assessment of ANS arousal and HPA axis reactivity via salivary cortisol. No previous studies have included a standardized assessment of visceral (water load) pain to examine outcomes following a mind-body intervention in young IBS patients.

The study will serve to broaden the body of research on the use of yoga to address chronic pain conditions. Young sufferers of IBS represent an especially important group to assist due to the likely longevity of their pain and limitations. The minimal side effects of IY, especially compared to other current treatments, make it attractive for a population who expect to be active in work, study, and recreation. The IY intervention- if found to be effective in improving IBS symptoms, quality of life, and reducing pain- could provide a self-help tool for long-term use for young people with IBS. Preliminary findings from this study will inform larger-scale investigations by determining effect size and thus sample sizes needed for future multi-center studies. The results of this research will yield initial data needed as a pre-requisite to a larger level evaluation of an IY intervention for IBS in adolescents and young adults including estimates of intervention parameters (attrition rates, response rates, confidence interval estimates of effect size), variability in outcomes, willingness to accept randomization, and potential sampling biases.

If shown in this study to significantly improve symptoms and functioning, further research could examine additional psychobiological mechanisms that might be responsible for these changes. For example, future studies should examine systemic pro-inflammatory cytokines and/or a range of stress hormones that might relate to changes in gut functioning, physical function, and mood. IY has practical, heuristic value in that it can be taught by the many trained teachers of IY once the protocol has been established in this study, with such applicability allowing broad access to this intervention. Finally, by providing information regarding an evidenced-based intervention for IBS, data from a subsequent multi-centered IBS RCT may help support health insurance coverage of specific yoga programs for patients with IBS.

## List of abbreviations

ANCOVA: analysis of covariance; ANS: autonomic nervous system; BSI-18: Brief Symptom Inventory 18; CPAQ: Chronic Pain Acceptance Questionnaire; CRH: corticotropin releasing hormone; CSI: Child Somatization Inventory; FDI: Functional Disability Index; GI: gastrointestinal; GIS: Global Improvement Scale; HMO: Health Maintenance Organization; HPA: hypothalamic-pituitary-adrenal; HR: heart period; HRQOL: health-related quality of life; HRV: heart rate variability; IBS: Irritable Bowel Syndrome; IBS-SSS: IBS severity scoring system; ITT: intent-to-treat; IY: Iyengar yoga; LMP: last menstrual period; MAAS: Mindfulness Attention Awareness Scale; MIRB1: Medical Institutional Review Board 1; PCS: Pain Catastrophizing Scale; PPP: Pediatric Pain Program; PSEQ: Pain Self-Efficacy Questionnaire; RAP: recurrent abdominal pain; RCADS: Revised Child Anxiety and Depression Scale; RCT: randomized controlled trial; RSA: respiratory sinus arrhythmia; SC: skin conductance; SCAS: Spence Children's Anxiety Scale; SF-36: Short Form-36; SIBO: small intestinal bacterial overgrowth; VAS: visual analog scale; VSI: Visceral Sensitivity Index; WLT: Water Load Task.

## Competing interests

The authors declare that they have no competing interests.

## Authors' contributions

SE, JT and LZ participated in the conception of the trial and in plans for the data analysis. SE, JT, LZ and LC drafted the manuscript. All authors read and approved the final manuscript.
